# Regulation of Hippocamposeptal Synaptic Transmission by GABA_B_Rs Is Altered in 5XFAD Mice in a Sex- and Age-Dependent Manner

**DOI:** 10.1007/s12031-024-02260-0

**Published:** 2024-08-30

**Authors:** Joanne C. Damborsky, Jerrel L. Yakel

**Affiliations:** Neurobiology Laboratory, National Institute of Environmental Health Sciences, National Institutes of Health, Department of Health and Human Services, 111 TW Alexander Dr., P.O. Box 12233, Mail Drop F2-08, Research Triangle Park, NC 27709 USA

**Keywords:** Hippocamposeptal, Medial septum, Alzheimer’s disease, GABA, GABA_B_

## Abstract

Hippocamposeptal (HS) neurons send GABAergic projections from the hippocampus to the medial septum/diagonal band of Broca (MS/DBB) as part of a reciprocal loop that is critical for memory. HS neurons are proposed to be particularly sensitive to the deleterious effects of pathological exposure to amyloid-β (Aβ), as would occur during Alzheimer’s disease (AD). However, it is not known how HS GABA release in the MS/DBB is altered during the progression of AD. To target HS neurons in a mouse model of AD, we crossed SST-Cre mice to 5XFAD mice and performed stereotaxic injections of Cre-dependent AAV containing mCherry/channelrhodopsin-2 (ChR2) into the hippocampus of offspring at 4, 6, 9, and 12 months. We used optogenetics to selectively stimulate HS terminals while performing whole-cell patch-clamp recordings from MS/DBB neurons in slices. There was a transient reduction in HS-inhibitory postsynaptic current (IPSC) amplitude in female 5XFAD mice at 6 months, but no difference in males at any age, and no difference in paired-pulse ratio in either sex at any age. When bath applying the GABA_B_R agonist, baclofen, we found a larger decrease in HS-IPSC amplitude in 5XFAD females at 9 months and 5XFAD males at 12 months. In 12-month-old 5XFAD females, response to baclofen was significantly reduced. These data suggest that there is a transient increase in responsiveness to GABA_B_R activation in 5XFAD mice that occurs earlier in females than in males. These sex-specific changes to HS function are likely to impact the relay of information between the hippocampus and MS/DBB.

## Introduction

Hippocamposeptal (HS) neurons are GABAergic neurons in the hippocampus that send projections to the medial septum/diagonal band of Broca (MS/DBB) (Alonso and Kohler 1982; Toth et al. 1993; Jinno and Kosaka 2002). HS neurons have extensive connections in both the hippocampus and MS/DBB (Toth et al. 1993; Blasco-Ibanez and Freund 1995; Gulyas et al. 2003; Takacs et al. 2008; Mattis et al. 2014; Yuan et al. 2017), making them uniquely positioned to modulate and synchronize activity between these two regions (Dragoi et al. 1999; Manseau et al. 2008; Quilichini et al. 2012; Kang et al. 2017). The MS/DBB and hippocampus, and connections between these regions, have been shown to play vital roles in regulating learning and memory (Mizumori et al. 1990; Burgess et al. 2002; Bannerman et al. 2004; Khakpai et al. 2013; Ballinger et al. 2016; Haam and Yakel 2017). Thus, it is likely that alterations to HS signaling may be associated with cognitive decline in diseases that affect memory, such as Alzheimer’s disease (AD). A loss of cholinergic signaling within the reciprocal septohippocampal pathway has long been associated with deficits in hippocampal-mediated memory formation and has been linked to AD (Davies and Maloney 1976; Perry et al. 1977; Bartus et al. 1982; Ballinger et al. 2016; Haam and Yakel 2017). However, less is known about how the function of HS neurons is affected during the pathogenesis of AD, leaving an incomplete picture of how septo-hippocampo-septal circuit activity is altered by AD.

Previous studies suggest that exposure to high levels of amyloid-β (Aβ) can lead to a loss of HS neurons (Villette et al. 2012; Kim et al. 2019). In the 5XFAD mouse model of AD, which displays rapid Aβ pathology (Oakley et al. 2006), there is a significant reduction in the density of HS fibers targeting MS neurons (Kim et al. 2019). HS neurons are also damaged if Aβ is directly infused into the hippocampus, and this has been linked to memory impairment along with a decrease in the firing rate of parvalbumin-expressing GABAergic neurons in the MS/DBB (Villette et al. 2010, 2012). While these studies point to a loss of HS neurons caused by Aβ, it is not known how this translates into changes in HS GABA release in response to pathological Aβ exposure. Understanding how HS GABA release in the MS/DBB is affected by Aβ will provide necessary information for understanding how activity within the larger septo-hippocampo-septal loop is altered in AD.

In addition to affecting baseline levels of HS GABA release, it is also possible that AD disrupts the modulation of HS GABA release by altering local circuitry and presynaptic receptors found on HS terminals. One receptor that has been shown to have a critical role for regulating HS GABA release in the MS/DBB is the GABA_B_R. Activation of GABA_B_Rs can directly and dramatically reduce HS GABA release (Damborsky and Yakel 2021). This regulation can further mediate the effects of other neuromodulatory signaling pathways that alter local inhibitory activity in the MS/DBB. Specifically, any activity that increases GABAergic signaling in the MS/DBB might activate GABA_B_Rs on HS terminals, thereby decreasing HS GABA release. This effect has been seen with cholinergic signaling, which increases spontaneous GABA release in the MS/DBB (Damborsky et al. 2017) and indirectly decreases HS GABA release through a GABA_B_R-dependent mechanism (Damborsky and Yakel 2021). There is still much to be learned about how GABA_B_Rs are affected in AD. In the hippocampus, early human studies indicated a decrease in GABA_B_R binding in multiple subregions (Chu et al. 1987). However, subsequent studies indicate that GABA_B_R1 expression may be increased in moderate AD and then decreased in severe AD (Iwakiri et al. 2005). It is not yet known how GABA_B_R modulation of HS GABA release is affected in AD, but any changes are likely to have far-reaching impacts on HS signaling.

Here, we examine how HS GABA release is altered in 5XFAD male and female mice and determine if GABA_B_R modulation of HS GABA release is affected. We determine that the magnitude and regulation of HS GABA release in the MS/DBB are altered in 5XFAD mice in a sex-specific manner. These findings provide insight into the dysfunction of signaling in the septo-hippocampo-septal circuit in AD and underline the importance of considering sex as an important variable in AD studies.

## Material and Methods

### Animals

Female homozygous SST-IRES-Cre (SST-Cre) mice (Taniguchi et al. 2011) (STOCK Sst < tm2.1(cre)Zjh > /J; RRID:IMSR_JAX:013044) were crossed to male hemizygous 5XFAD mice (Oakley et al. 2006) (6SJL-Tg(APPSwFlLon,PSEN1*M146L*L286V)6799Vas/Mmjax; RRID:MMRRC_034840-JAX). Offspring were all heterozygous for SST-Cre and either WT or hemizygous for 5XFAD mutations. Mice that were WT for the 5XFAD mutations were used as littermate controls. Mice were maintained on a 12-h light/dark cycle under constant temperature control with ad libitum access to food and water. All procedures were approved and performed in compliance with the NIEHS/NIH Humane Care and Use of Animals in Research protocols.

### Stereotaxic Injections

Stereotaxic injections were performed 2–3 weeks prior to the mice turning 4, 6, 9, or 12 months of age as previously described (Damborsky and Yakel 2021). Briefly, mice were injected with pAAV-EF1a-double floxed-hChR2(H134R)-mCherry-WPRE-HGHpA (Addgene plasmid #20297 from Karl Deisseroth), which was packaged with AAV stereotype 5 by the Viral Vector Core facility at the NIEHS. Mice were anesthetized and placed in a stereotaxic apparatus (Stoelting/Neurostar). Of virus, 0.5 µl (4 × 10^12^–1.5 × 10^13^ GC/ml) was infused bilaterally into the hippocampi at each of four different hippocampal coordinates (relative to Bregma): ± 1.6 ML, − 2 AP, 1.2, and 1.8 DV. Virus infusion rate was 0.1 μl/min. Previous data has shown that injection into these coordinates in SST-Cre mice results in robust expression in the hippocampus and fiber expression in the MS/DBB (Damborsky and Yakel 2021). Following surgery, mice were allowed to recover on a heating pad until ambulatory and monitored for signs of distress.

### Electrophysiology

Whole-cell patch-clamp recordings were performed as previously described (Damborsky and Yakel 2021). Briefly, mice were decapitated under deep anesthesia, the brain was removed, and 300-μm slices were taken through the MS/DBB while in ice-cold cutting solution containing (in mM) 230 sucrose, 3 KCl, 1.25 NaH_2_PO_4_, 28 NaHCO_3_, 5 glucose, 0.5 CaCl_2_, 7 MgCl_2_, 1 ascorbate, and 3 sodium pyruvate. In order to confirm proper targeting of virus to the hippocampus, hippocampal slices (300 µM) were taken from each mouse and examined for fluorescence using the same Axio Examiner A.1 (ZEISS) microscope used for electrophysiological recordings. The mCherry signal was activated by delivering 570-nm LED light generated using a CoolLED system delivered through the microscope objective and imaged using Andor iQ software (Oxford Instruments). Whole-cell recordings were performed with the slices in an artificial cerebrospinal fluid (ACSF) solution containing (in mM) 126 NaCl, 3.5 KCl, 1.2 NaH_2_PO_4_, 25 NaHCO_3_, 11 glucose, 2 CaCl_2_, and 1.3 MgCl_2_ that was continuously oxygenated with 95%O_2_/5%CO_2_. Patch pipettes were pulled to a resistance of 3–7 MΩ using P-97 horizontal puller (Sutter Instrument Co.) and were filled with an internal pipette solution that contained (in mM) 130 K^+^-gluconate, 10 KCl, 10 HEPES, 1 EGTA, 2 MgCl_2_, 3 MgATP, and 0.3 NaGTP with pH 7.2–7.3 and osmolarity 280–290 mOsm. Cells were held at − 80 mV, and were only used for analysis if access resistance was below 40 MΩ and varied by less than 20% throughout the experiment. Inhibitory postsynaptic currents (IPSCs) were isolated by bath application of 10 μM DNQX and 40 μM AP5 to block AMPA and NMDA receptors, respectively. Activation of ChR2 was achieved using an Andor Mosaic 3 illumination system (Oxford Instruments), with 460-nm blue LED light generated using a CoolLED system delivered through the microscope objective. Light pulses (5 ms) were given at the lowest possible intensity of 1% maximum power, which corresponded to approximately 0.2 mW as measured by a PM100D power meter (Thorlabs). Andor iQ software was used to control the delivery of light. The paired-pulse stimulation protocol was repeated three to four times with 20-s intervals between runs, and the average of these traces was used for data analysis. HS-IPSC amplitude was measured as the difference in the current of the first peak relative to baseline. Paired-pulse ratios were not calculated if the amplitude of either IPSC was 0. The recording chamber was continuously perfused with oxygenated ACSF at a rate of approximately 2 ml/min, and drugs were allowed to be washed on for at least 3 min to ensure complete exchange of bath fluid and slice penetration. Recordings were digitized using an Axon Digidata 1550 digitizer (Molecular Devices) and collected using pClamp 10 software (Molecular Devices) with a Multiclamp 700B amplifier (Molecular Devices). Data were filtered at 2 kHz and sampled at 10 kHz. AP5, DNQX, and Baclofen were acquired from Tocris Bioscience.

### Statistical Analyses

Electrophysiology data was analyzed using pClamp 10 software. SigmaPlot 14 (Systat Software, Inc.) was used for statistical analysis, and GraphPad Prism 10 was used for graphical illustrations. Within-group analysis was used to compare responses from the same cell prior to and following baclofen exposure. Data were tested for normality using as Shapiro–Wilk test and then analyzed using a paired *t*-test or Wilcoxon signed-rank test, as appropriate. Between-group analysis to compare responses across ages and genotypes was performed using a two-way ANOVA. Planned Bonferroni multiple comparisons were performed based on a priori hypotheses to compare differences between genotypes at each age and between ages within each genotype. Significance was defined as a *p* value of < 0.05. The number of cells recorded is reported as *n* values. At least six mice were used in each group. Data are presented ± S.E.M.

## Results

**Fig. 1:**
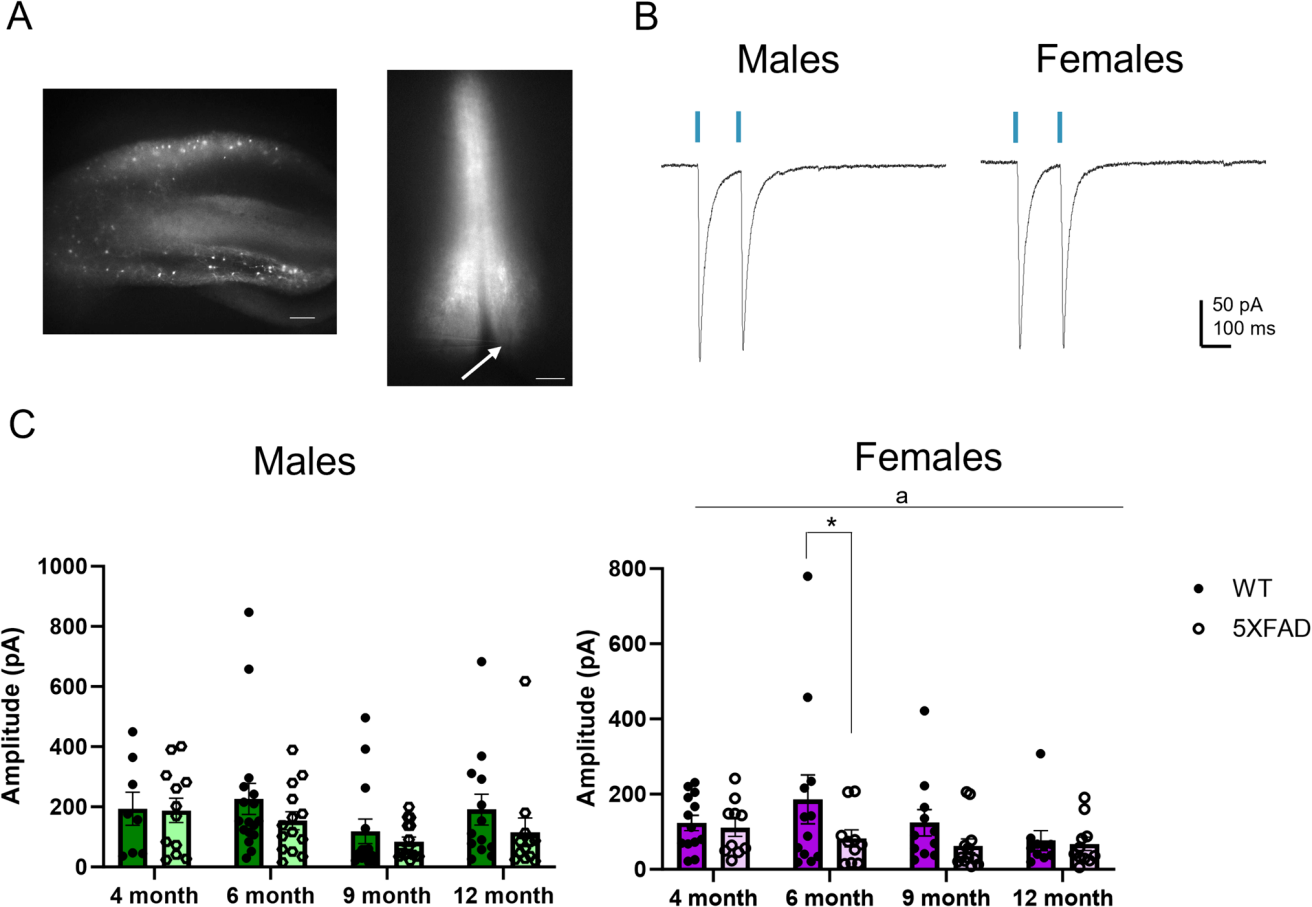
HS-IPSC amplitudes in WT and 5XFAD mice. **A** Representative images showing fluorescent expression in the hippocampus and MS/DBB of an injected 5XFAD × SST-Cre mouse. The arrow denotes the tip of a recording pipette from a whole-cell recording in the DBB. Scale bar = 200 µM. **B** Representative averaged trace showing HS-IPSC following brief (5 ms) blue light pulses in male and female mice. **C** Amplitude of the first ISPC in a paired-pule protocol in male WT and 5XFAD mice at 4 (*n* = 8, 12), 6 (*n* = 17, 14), 9 (*n* = 12, 15), and 12 (*n* = 13, 12) months and amplitude of the first ISPC in a paired-pule protocol in female WT and 5XFAD mice at 4 (*n* = 13, 10), 6 (*n* = 12, 10), 9 (*n* = 11, 13), and 12 (*n* = 10, 11) months. Data analyzed using a two-way ANOVA with planned Bonferroni multiple comparisons. Data shown ± S.E.M. *p < 0.05; a, overall genotype effect *p* < 0.05

In order to assess how HS GABA release in the MS/DBB may be affected by AD-like pathology, we sought to selectively stimulate and record HS synaptic transmission in the 5XFAD mouse model of AD (Oakley et al. 2006). To do this, we stereotaxically injected AAV5-EF1a-double floxed-hChR2(H134R)-mCherry-WPRE-HGHpA bilaterally into the hippocampi of SST-Cre::5XFAD mice. This resulted in selective expression of ChR2 and mCherry in SST + neurons in the hippocampus and HS fiber expression in the MS/DBB as previously described for SST-Cre mice (Damborsky and Yakel 2021) (Fig. 1A). To compare changes in HS-IPSC in 5XFAD and WT mice, mice that were heterozygous for SST-Cre and hemizygous for 5XFAD mutations (5XFAD) were compared to littermates that were heterozygous for SST-Cre and negative for 5XFAD mutations (WT). Mice were injected 2–3 weeks prior to whole-cell recordings, which occurred at 4, 6, 9, and 12 months of age. During whole-cell recordings from MS/DBB neurons made in the presence of DNQX and AP5, a brief (5 ms) blue light (460 nm) pulse resulted in an HS-mediated IPSC (HS-IPSC) (Fig. 1B), confirming the presence of ChR2 in GABAergic HS fibers synapsing onto MS/DBB neurons.

**Fig. 2:**
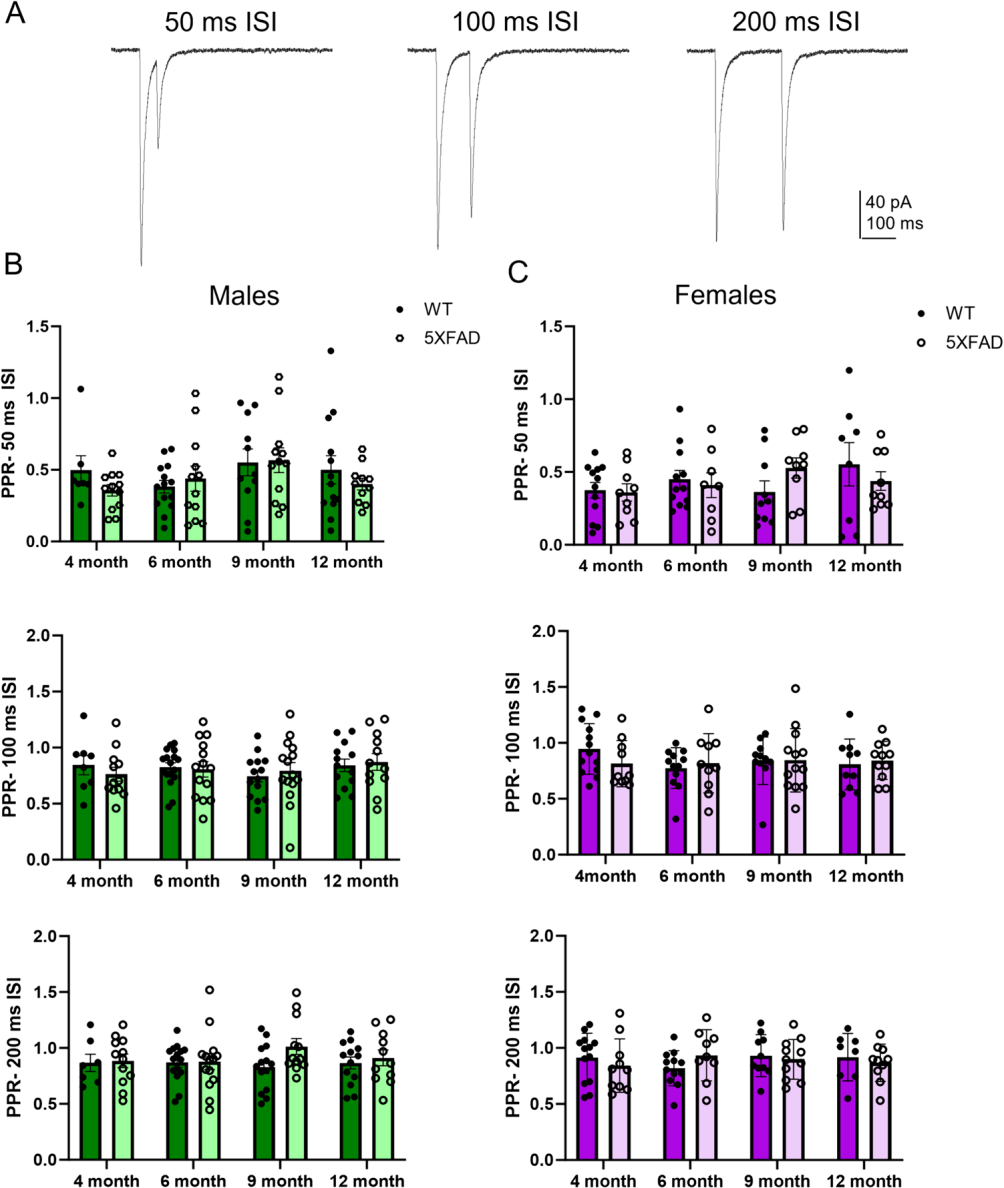
HS-IPSC paired-pulse ratios in WT and 5XFAD mice. **A** Representative averaged traces showing HS-IPSCs following brief (5 ms) blue light pulses separated by 50, 100, or 200 ms. **B** Comparisons of PPR in male WT and 5XFAD mice across ages at the 50-, 100-, and 200-ms inter-stimulus interval (ISI) (*n* = 7 to 17 cells). **C** Comparisons of PPR in female WT and 5XFAD mice across ages at the 50-, 100-, and 200-ms ISI (*n* = 8 to 13 cells). Data analyzed using a two-way ANOVA with planned Bonferroni multiple comparisons. Data shown ± S.E.M

Two different measurements were used to assess how HS GABA release is affected by aging in WT and 5XFAD mice. During whole-cell recordings, two pulses of light were given, resulting in two HS-IPSCs. We first measured the amplitude of the first IPSC to compare the magnitude of HS GABA release. Secondly, we also compared the ratio of the amplitude of the second IPSC to the ratio of the first IPSC, known as the paired-pulse ratio (PPR), to assess presynaptic release probability. When we compared the amplitude of male WT and 5XFAD mice across ages, we found no significant difference (Fig. 1C). Conversely, in females, there was an overall genotype effect, with 5XFAD having significantly smaller HS-IPSC amplitude than WT (*p* = 0.049; Fig. 1C). Bonferroni comparisons indicated that this effect was largest at 6 months of age, when 5XFAD mice had significantly smaller HS-IPSC amplitudes than WT mice (*p* = 0.031). For PPR analysis, we used three different inter-stimulus intervals (ISI), defined as the time between the start of the first 5-ms pulse to the time of the start of the second 5-ms pulse: 50 ms, 100 ms, and 200 ms (Fig. 2A). There was no significant difference at an any age in either males or females between 5XFAD and WT mice at any of the three ISIs (Fig. 2B and C), indicating that presynaptic release probability of HS neurons is unchanged in 5XFAD mice compared to WT littermates.

**Fig. 3:**
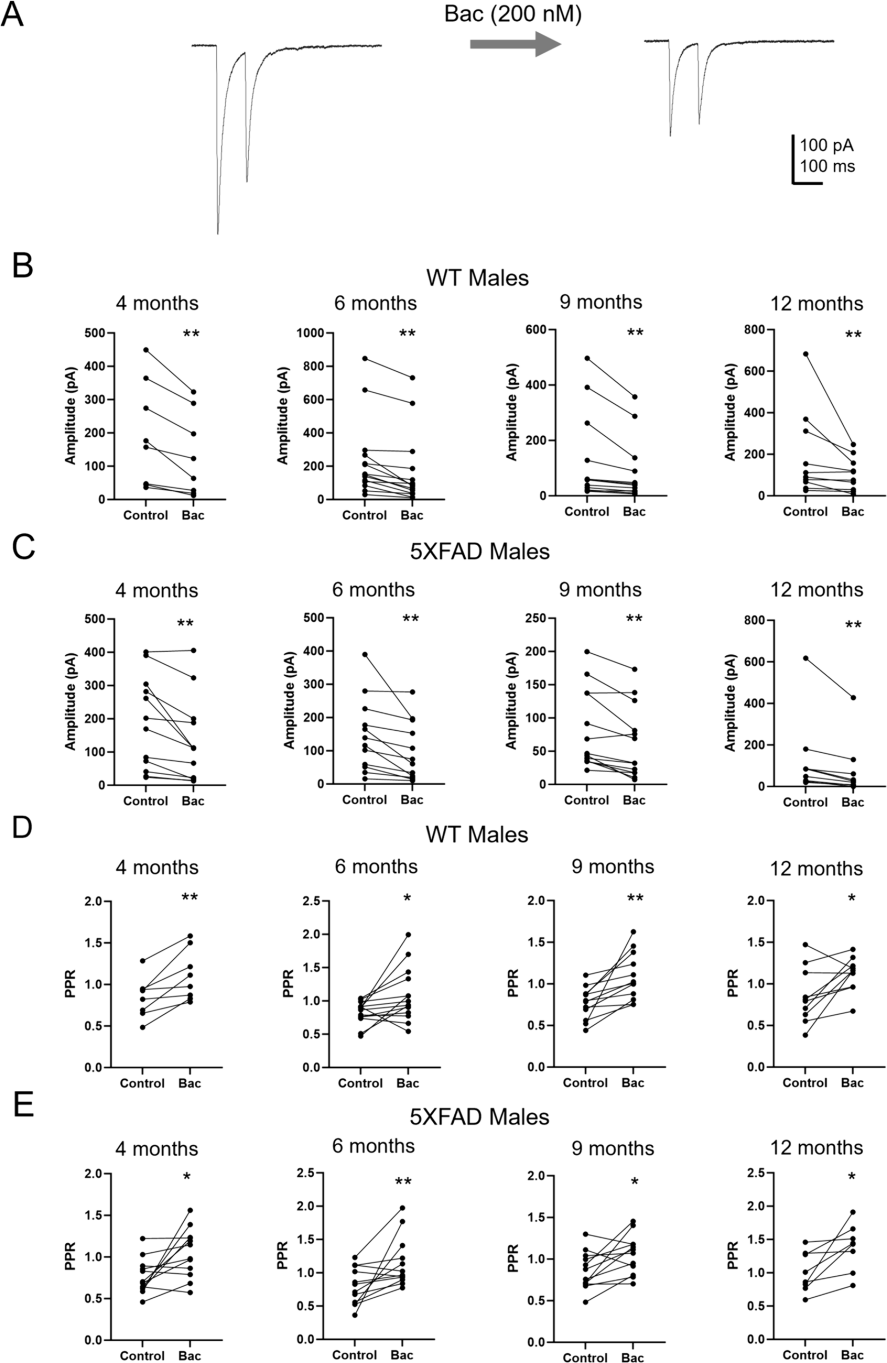
Effects of baclofen on HS-IPSCs in male mice. **A** Representative averaged traces showing HS-IPSCs with 100-ms ISI prior to and following bath application of baclofen (200 nM). **B** Amplitude of the first IPSC in male WT mice prior to and following bath application of baclofen at 4 (*n* = 8), 6 (*n* = 14), 9 (*n* = 12), and 12 (*n* = 10) months. **C** Amplitude of the first IPSC in male 5XFAD mice prior to and following bath application of baclofen at 4 (*n* = 12), 6 (*n* = 12), 9 (*n* = 13), and 12 (*n* = 9) months. **D** Paired-pulse ratio of HS-IPSCs in male WT mice prior to and following batch application of baclofen at 4 (*n* = 8), 6 (*n* = 14), 9 (*n* = 12), and 12 (*n* = 10) months. **E** Paired-pulse ratio of HS-IPSCs in male 5XFAD mice prior to and following batch application of baclofen at 4 (*n* = 12), 6 (*n* = 12), 9 (*n* = 12), and 12 (*n* = 8) months. Data analyzed by a paired *t*-test or Wilcoxon signed-rank test based on normality. Data shown ± S.E.M. *p < 0.05, **p < 0.01

**Fig. 4:**
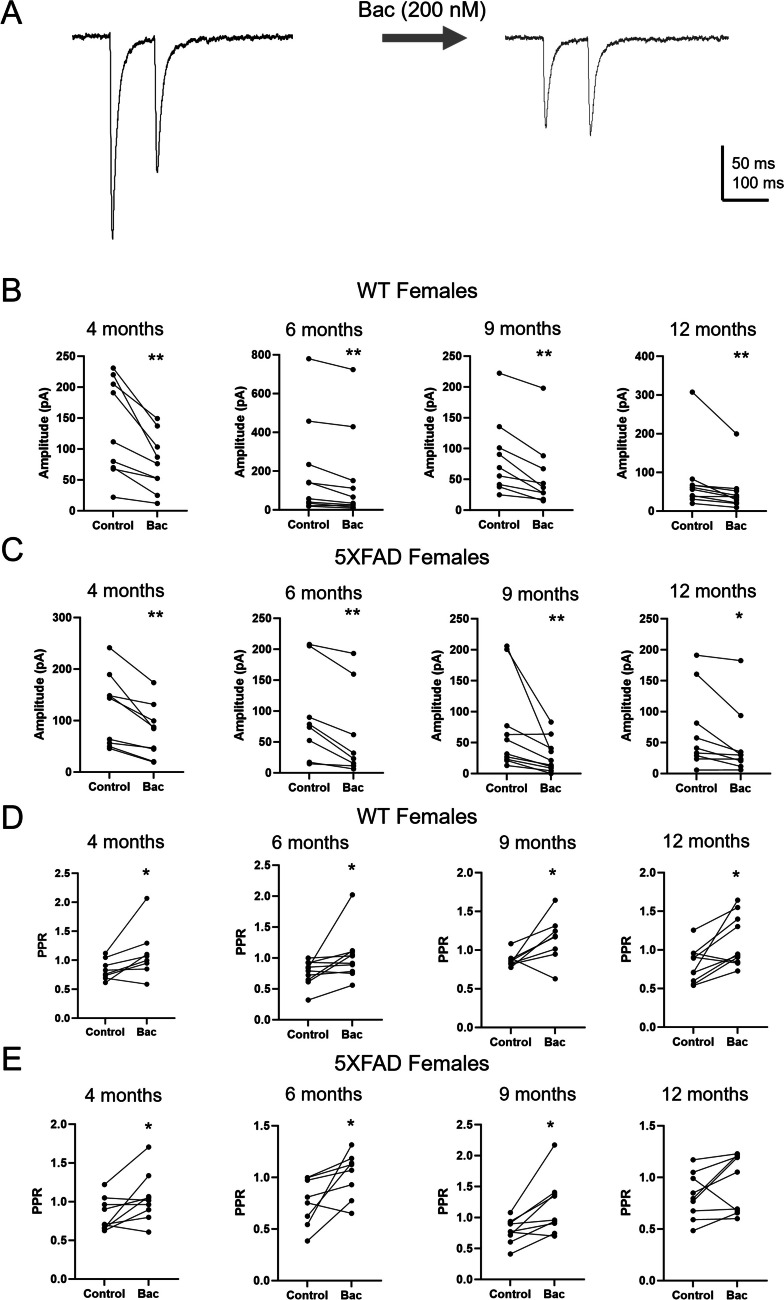
Effects of baclofen on HS-IPSCs in female mice. **A** Representative averaged traces showing HS-IPSCs with 100-ms ISI prior to and following bath application of baclofen (200 nM). **B** Amplitude of the first IPSC in female WT mice prior to and following bath application of baclofen at 4 (*n* = 9), 6 (*n* = 10), 9 (*n* = 9), and 12 (*n* = 10) months. **C** Amplitude of the first IPSC in female 5XFAD mice prior to and following bath application of baclofen at 4 (*n* = 9), 6 (*n* = 8), 9 (*n* = 10), and 12 (*n* = 9) months. **D** Paired-pulse ratio of HS-IPSCs in female WT mice prior to and following batch application of baclofen at 4 (*n* = 8), 6 (*n* = 10), 9 (*n* = 8), and 12 (*n* = 10) months. **E** Paired-pulse ratio of HS-IPSCs in female 5XFAD mice prior to and following batch application of baclofen at 4 (*n* = 9), 6 (*n* = 8), 9 (*n* = 9), and 12 (*n* = 9) months. Data analyzed by a paired *t*-test or Wilcoxon signed-rank test based on normality. Data shown ± S.E.M. *p < 0.05, **p < 0.01

We previously found that HS GABA release is decreased by presynaptic GABA_B_R activation (Damborsky and Yakel 2021). In order to determine if this regulation of HS GABA release is altered in 5XFAD mice, a low dose (200 nM) of the GABA_B_R agonist baclofen was bath applied to slices during recordings. In comparison to high concentrations of baclofen (20 μM), which completely block HS GABA release, this low concentration of baclofen has been shown to reduce, but not block, HS-IPSC amplitude and increase HS-IPSC PPR (Damborsky and Yakel 2021). We therefore compared HS-IPSC amplitude and PPR at the 100-ms ISI prior to and following baclofen wash-on (Figs. 3A and 4A). In male WT mice, baclofen significantly reduced HS-IPSC amplitude at 4 (*p* = 0.004), 6 (*p* = 0.0003), 9 (*p* = 0.001), and 12 (*p* = 0.006) months (Fig. 3B). Baclofen also increased PPR in male WT mice at 4 (*p* = 0.005), 6 (*p* = 0.025), 9 (*p* = 0.001), and 12 (*p* = 0.029) months (Fig. 3D). In male 5XFAD mice, baclofen significantly reduced HS-IPSC amplitude at 4 (*p* = 0.001), 6 (*p* = 0.001), 9 (*p* = 0.002), and 12 (*p* = 0.004) months (Fig. 3C). It also increased PPR in male 5XFAD mice at 4 (*p* = 0.015), 6 (*p* = 0.002), 9 (*p* = 0.02), and 12 (*p* = 0.021) months (Fig. 3E). In female WT mice, baclofen significantly reduced HS-IPSC amplitude at 4 (*p* = 0.004), 6 (*p* = 0.005), 9 (*p* = 0.0008), and 12 (*p* = 0.004) months (Fig. 4B). It also increased PPR in female WT mice at 4 (*p* = 0.046), 6 (*p* = 0.014), 9 (*p* = 0.04), and 12 (*p* = 0.01) months (Fig. 4D). In female 5XFAD mice, baclofen decreased HS-IPSC amplitude at 4 (*p* = 0.003), 6 (*p* = 0.003), 9 (*p* = 0.004), and 12 (*p* = 0.026) months (Fig. 4C). It also increased PPR in female 5XFAD mice at 4 (*p* = 0.038), 6 (*p* = 0.032), and 9 (*p* = 0.01) months. There was no significant effect of baclofen on PPR in 12-month-old female 5XFAD mice (*p* = 0.13) (Fig. 4E).

**Fig. 5:**
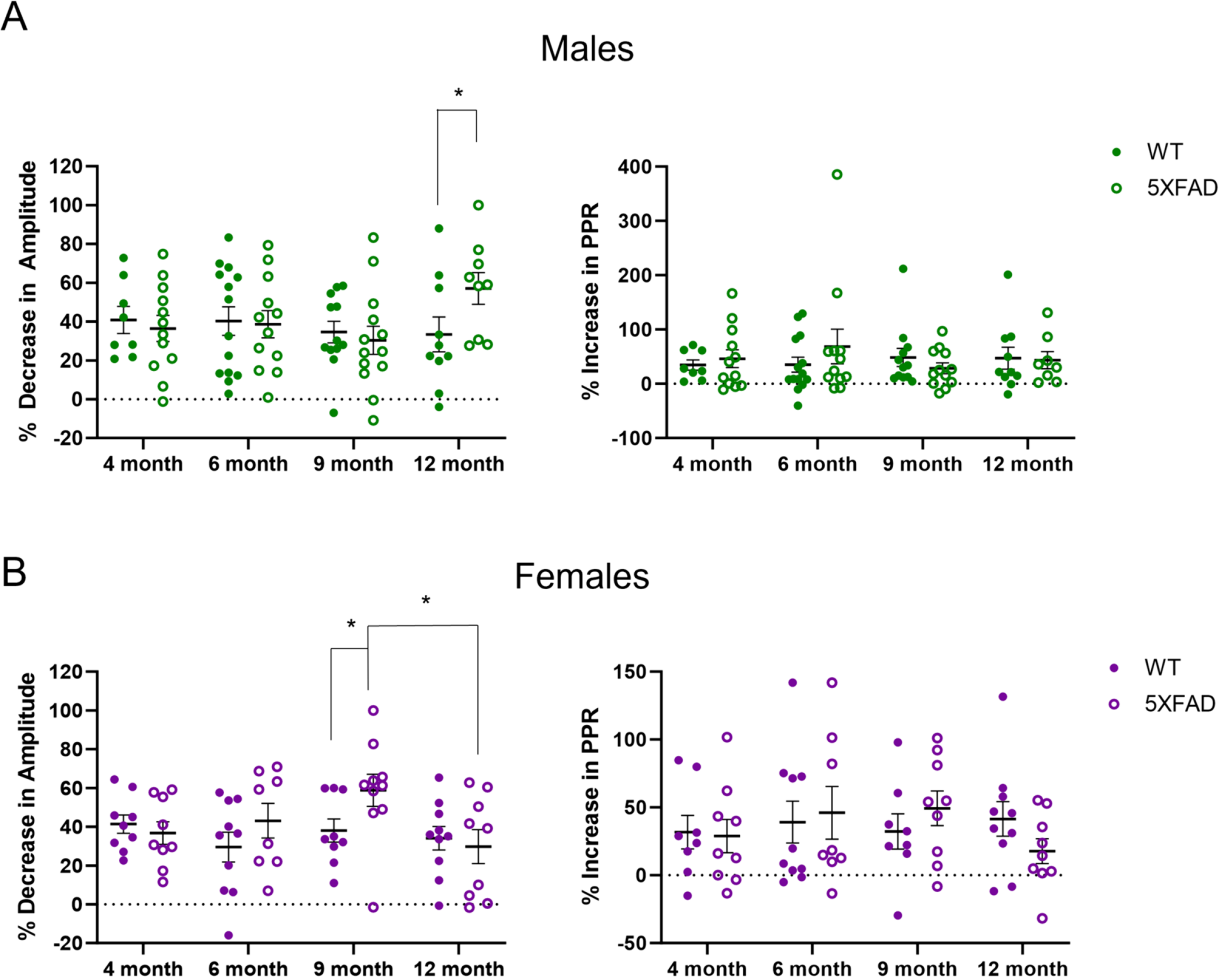
Effects of baclofen across ages and genotypes. **A** Comparison of the percent decrease in amplitude and percent increase in PPR of HS-IPSCs following bath application of baclofen in male WT and 5XFAD mice across ages. **B** Comparison of the percent decrease in amplitude and the percent increase in PPR of HS-IPSCs following bath application of baclofen in female WT and 5XFAD mice across ages. Data analyzed using a two-way ANOVA with planned Bonferroni multiple comparisons. Data shown ± S.E.M. *N* = 8–14 cells; *p < 0.05

To compare the magnitude of the effect of baclofen in WT and 5XFAD mice across ages, we compared the percent decrease in amplitude and the percent increase in PPR across all groups. In males, 12-month-old 5XFAD mice had a significantly greater decrease in amplitude compared to 12-month WT mice following baclofen exposure (*p* = 0.039; Fig. 5A). There were no significant differences in the percent increase in PPR. In females, 9-month-old 5XFAD mice had a significantly greater decrease in amplitude compared to both 9-month WT mice (*p* = 0.042) and 12-month 5XFAD mice (*p* = 0.031; Fig. 5B). There were no significant differences in the increase in PPR. Thus, in female 5XFAD mice, there was a transient increase in response to GABA_B_R activation at 9 months, whereas in males, this increase in response is not seen until 12 months.

## Discussion

Understanding how activity within the septo-hippocampo-septal loop is altered in AD is vital for connecting dysfunction within this circuit to cognitive impairment and determining potential targets for therapeutic interventions to alleviate these symptoms. Despite their critical location within this loop, there is still little known about how HS GABA release is altered in AD. Here, we provide data to show that in female 5XFAD mice, there is a transient decrease in HS GABAergic transmission in the MS/DBB at 6 months of age, with no differences in males through 12 months of age. We also report sex-specific alterations in the regulation of HS GABA release by GABA_B_Rs, with females showing an increase in response to baclofen at 9 months and males not until 12 months. This effect in females was also transient in nature, as by 12 months, the response to baclofen was significantly reduced. These sex-specific, age-specific effects point to an HS system that is relatively functionally resilient in 5XFAD mice. Once functional changes begin to occur, they arise to a greater degree and at an accelerated timeline in female 5XFAD mice compared to their male counterparts. These findings highlight the fluidity of circuitry changes that might occur within the HS system during the progression of AD and highlight the importance of examining different timepoints in both males and females throughout aging to gain a better understanding of these changes.

5XFAD mice display rapid amyloid pathology, with memory impairments in various tasks reported as early as 4–6 months of age (Oakley et al. 2006; Kimura and Ohno 2009; Ohno 2009; Girard et al. 2014; Li et al. 2021). In the MS, intracellular Aβ accumulation can be seen at 2 months, and extracellular plaques are present by 6–7 months (Daini et al. 2021). In the hippocampus, neurons that express SST, including HS neurons, have been shown to be particularly vulnerable to the deleterious effects of Aβ (Ramos et al. 2006; Perez-Cruz et al. 2011; Villette et al. 2012; Jang et al. 2023). A decrease of HS connectivity from the dentate gyrus and subiculum to the MS has been observed as early as 4.5 months in 5XFAD mice, while a decrease of connectivity from CA1 and CA3 to MS was additionally observed at 14 months (Kim et al. 2019). Interestingly, here, we show that, in males, there is no difference in HS-IPSC amplitude through 12 months of age. In females, there was an overall effect of genotype on HS-IPSC amplitude. However, additional analysis revealed that the only age that was independently significantly different was 6 months. Additionally, there was no difference in HS-IPSC amplitude between 6 and 12 months of age. This indicates that while there was an initial decrease in HS GABA release in 5XFAD females, GABA release does not continue to deteriorate as AD-like pathology worsens. Additionally, our data show that there is no difference in PPR in 5XFAD mice, indicating that presynaptic release probability remains unchanged. These results suggest a remarkable stability of functional HS input in the MS/DBB even while Aβ levels, neuronal loss, and cognitive impairments mount.

The seemingly incongruous findings between structural studies and the functional data reported here could point to active compensatory mechanisms within the HS pathway to alleviate the effects of a loss of neuron number. Such functional compensation has previously been reported for GABAergic signaling in the hippocampus. In the GABAergic septohippocampal pathway, circuit remodeling counteracts the effects of GABAergic hyperactivity in the DG of 3xTg-AD mice (Wander et al. 2023). There is also a transient increase in GAD65 and GAT3 protein expression in the hippocampus of APP/PS1 mice at 6 months (Salazar et al. 2021), which could increase GABA synthesis and transport, potentially leading to an increase in GABA release. Outside of the GABAergic system, compensatory changes in postsynaptic receptor density have been reported in response to AD-like pathology. In the cholinergic system, an upregulation of nicotinic acetylcholine receptor signaling has been observed in the prefrontal cortex of two AD mouse models (Power et al. 2023). Similarly, in the glutamatergic system, a loss of excitatory synapse coincides with an increase in AMPA receptors in the CA1 hippocampus of 5XFAD mice (Neuman et al. 2015). At GABAergic synapses, fast inhibitory postsynaptic currents are mediated by GABA_A_Rs. While there is still debate about how the expression of these receptors changes in AD, the majority of studies point to either a decrease or no change in functional receptor expression along with changes in subunit composition in the hippocampus and cortex (Limon et al. 2012; Govindpani et al. 2017; Kwakowsky et al. 2018; Carello-Collar et al. 2023). However, less is known about how GABA_A_Rs are affected in the MS/DBB, and it is possible that upregulation of GABA_A_R signaling precedes a loss of receptors. The functional resilience of HS neurons reported here suggests that information flow from the hippocampus to MS/DBB persists at relatively consistent rates even while neuronal loss and dysfunction are occurring in both regions. This could have a profound effect on regulating and synchronizing activity between these two regions (Dragoi et al. 1999; Manseau et al. 2008; Quilichini et al. 2012; Kang et al. 2017). Understanding why HS projections remain functionally resilient even while neurons in the hippocampus and MS/DBB are lost will be key to understanding how AD pathology impacts communication between these two regions.

Dysregulation of the GABAergic system and perturbations to the balance of excitation and inhibition within the brain are likely to be contributing factors to cognitive decline in AD. (Nava-Mesa et al. 2014; Jimenez-Balado and Eich 2021). As regulators of presynaptic GABA release, GABA_B_Rs are linchpins of GABAergic signaling, and dysfunction of these receptors would have far-reaching effects on inhibitory circuits in the brain. However, there is still much uncertainty about how GABA_B_R function is altered in AD. In a recent study using APP/PS1 mice, GABA_B_R subunit protein expression and mRNA transcript levels were found to be decreased in the hippocampus as early as 6 months (Salazar et al. 2021). Conversely, another study found no change in GABA_B1_ protein expression in the hippocampus through 12 months in APP/PS1 mice but did note a reduction in surface levels of GABA_B_Rs (Martin-Belmonte et al. 2020). Incubation of hippocampal slices with Aβ_25-35_ did not affect mRNA levels of GABA_B1_ or GABA_B2_ receptor subunits, but did reduce expression of the subunits of the G protein-gated inwardly rectifying K^+^ (GIRK) channel that is activated by GABA_B_Rs (Mayordomo-Cava et al. 2015). Pre- and postsynaptic GIRK1 and 2 subunit density in the CA1 hippocampus is also decreased in APP/PS1 mice at 12 months (Martin-Belmonte et al. 2022), potentially leading to dysfunction of signaling through GABA_B_Rs. Early human studies have also found reduced numbers of GABA_B_Rs in the hippocampus of individuals with AD (Chu et al. 1987). Interestingly, while these animal and human studies mostly point to a decrease in GABA_B_R expression or signaling in the hippocampus as a result of Aβ exposure, our data show that there is a transient increase in the effects of baclofen, indicating an amplification of signaling through GABA_B_Rs. Subsequently, in 12-month-old females, there is a significant decrease in baclofen-induced reductions in HS-IPSC amplitude. This is also the only group that shows no change in PPR induced by baclofen, signifying a loss of presynaptic GABA_B_R signaling. These results are more in line with human data that has shown that there is a transient increase in the expression of GABA_B1_ subunits in the hippocampus in moderate AD, followed by a decrease in expression in severe AD (Iwakiri et al. 2005). Additionally, in rats, it has been shown that intrahippocampal injection of a GABA_B_R antagonist can protect against the memory impairments caused by acute Aβ toxicity (Almasi et al. 2018), suggesting that hyperactivity of the GABA_B_R may contribute to the cognitive deficits induced by Aβ. Together, these studies suggest that the function of GABA_B_Rs might be differentially altered depending on the degree of progression of AD-like pathology. This study contributes to accumulating evidence suggesting that signaling through GABA_B_Rs might initially increase in AD and then decrease as the disease progresses. Understanding the exact timing and mechanisms of this duality will be critical for understanding if there is a potential for therapeutic benefit via modulation of GABA_B_Rs.

One of the most striking findings of this study is the divergence of effects on HS projections in females versus males. In addition to the female-specific reduction in HS amplitude in 5XFAD mice, we also saw a sex dependence of the timing of increase in the response to baclofen. In females, the increase is seen at 9 months, whereas in males, the increase is not seen until 12 months. Twelve-month-old 5XFAD females are also the only group that did not have a significant increase in PPR in response to baclofen, suggesting a loss of presynaptic GABA_B_R-mediated signaling. These sex-specific effects could be due to the sex dependence of the pathology of AD. Nearly two-thirds of individuals with AD are women (Alzheimer’s A, 2019). While there may be confounding factors that contribute to this difference, such as the extended lifespan of females, there is also evidence that women with AD may experience more severe cognitive impairment than their male counterparts (Laws et al. 2018). There have also been sex differences observed in 5XFAD mice, with females exhibiting characteristics indicative of a more severe phenotype, including more severe molecular pathology, increased anxiety, increased expression of inflammatory markers, more pronounced changes in gene expression, and stress-induced increases in Aβ (Devi et al. 2010; Bundy et al. 2019; Oblak et al. 2021; Sil et al. 2022). Adding to these studies, our results suggest that females have substantial functional changes in HS release not seen in males and an accelerated timeline of dysfunction in GABA_B_R signaling within the HS pathway, further suggesting a more severe AD phenotype.

In conclusion, we show that HS synaptic transmission is transiently reduced in female 5XFAD mice at 6 months and remains unchanged in 5XFAD males through 12 months. There is also a transient increase in response to GABA_B_R activation in 5XFAD females at 9 months and a loss of presynaptic regulation by GABA_B_Rs at 12 months. In 5XFAD males, an increase in GABA_B_R signaling was observed at 12 months. Many of the differences in HS GABA release and regulation do not occur until the later stages of AD-like pathology, and are transient in nature, pointing to potential compensatory mechanisms. These alterations are also sex-specific, with females having larger differences and an accelerated timeline of dysfunction compared to males. These data add a critical component of understanding to the question of how activity within the septo-hippocampo-septal loop is affected in AD.

## Data Availability

Data will be made available upon request.
